# Blue and green luminescent carbon nanodots from controllable fuel-rich flame reactors

**DOI:** 10.1038/s41598-019-50919-1

**Published:** 2019-10-10

**Authors:** Carmela Russo, Barbara Apicella, Anna Ciajolo

**Affiliations:** Istituto di Ricerche sulla Combustione, Consiglio Nazionale delle Ricerche, Piazzale V. Tecchio 80, 80125 Napoli, Italy

**Keywords:** Quantum dots, Structural properties

## Abstract

The continuous synthesis in controlled gas flame reactors is here demonstrated as a very effective approach for the direct and easy production of structurally reproducible carbon nanodots. In this work, the design of a simple deposition system, inserted into the reactor, is introduced. A controlled flame reactor is employed in the present investigation. The system was optimized for the production of carbon nanoparticles including fluorescent nanocarbons. Blue and green fluorescent carbon could be easily separated from the carbon nanoparticles by extraction with organic solvents and characterized by advanced chemical (size exclusion chromatography and mass spectrometry) and spectroscopic analysis. The blue fluorescent carbon comprised a mixture of molecular fluorophores and aromatic domains; the green fluorescent carbon was composed of aromatic domains (10–20 aromatic condensed rings), bonded and/or turbostratically stacked together. The green-fluorescent carbon nanodots produced in the flame reactor were insoluble in water but soluble in N-methylpyrrolidinone and showed excitation-independent luminescence. These results provide insights for a simple and controlled synthesis of carbon nanodots with specific and versatile features, which is a promising pathway for their use in quite different applicative sectors of bioimaging.

## Introduction

The new class of luminescent quantum dots named carbon dots (CDs) has drawn a lot of attention since the discovery of carbon fluorescent nanoparticles into arc discharge soot in 2004^1^ and the first attempt of synthesis^2^. CDs are eco-friendly candidates aiming at replacing both the semiconductor quantum nanodots and the organic dyes for biolabeling and bioimaging, as they offer advantages in terms of their low toxicity, biocompatibility and chemical stability. Curiously, just in the same year of CDs discovery, 2004, graphene was discovered as well^3^, and since then much effort has also been made to use graphene for producing graphene quantum dots (GQD)^4–7^. The CD is nowadays used as an umbrella term encompassing diverse classes of carbons, referred to with a variety of labels and acronyms (CD, CQD, GQD), having in common small (nanometric) sizes and specific luminescence properties. The research in the CDs field is now very active in looking for cheaper and high-yield CDs sources as demonstrated by the steeply growing literature, so vast to be continuously updated in many general survey articles see e.g.^4–16^.

Top-down CDs synthesis routes often involve harsh treatments as the oxidation of solid carbon precursors of quite different nature (e.g. graphite or carbon black), which is expensive due to the cost of both material and equipment, and is moreover time-consuming. Conversely, bottom-up synthesis methods based on the dehydration and the thermal carbonization of small organic molecules, biomass-based and synthetic polymers, food products, wastes are often cheaper, efficient and easily applicable on a large scale^8^. The different precursors employed and the empirical character of most of these bottom-up processes make the synthesis of these products difficult to be controlled and leading to CDs of variable polydispersity and properties.

It is worth noting that the carbonization reactions, necessary in bottom-up systems for the CDs formation, typically occur in combustion processes. Currently, the combustion systems have become topical for the chemical processing^17^ and the synthesis of inorganic and carbon materials. In particular, flame synthesis has been studied as a viable source of fullerenes, carbon nanotubes, and graphene-based materials^18–20^. Carbonization is favored by the lack of oxygen and the high temperature featuring fuel-rich combustion leading to the formation of carbon particulate matter mainly constituted of tarry organic carbon and solid carbon particles (soot). The latter occurs in a wide nanometric (individual particles) to micrometric (aggregates) size range. Notably, carbon particulate matter derived from combustion presents some common features as compared to CDs. First, the mixed amorphous-crystalline character of soot particles, observed especially in the initial inception phase of soot formation process^21^, traces just to CDs as amorphous and/or crystalline nanoparticles^22^. Second, small (two- to seven-membered rings) polycyclic aromatic hydrocarbons (PAH) individuated by chromatographic/mass spectrometric analysis of organic species extracted from soot^23–25^, are strongly fluorescent in the blue-violet^25–27^, recalling just the three- and four-membered ring PAH considered to mimic the optical properties of carbon dots^28,29^. The deep knowledge acquired in the field of soot formation can be applied to the topic of flame synthesis of carbon nanomaterials, providing a useful database for the use of combustion systems as controllable and tunable sources of light-absorbing and luminescent carbon. Because of environmental effects^30,31^, the formation of PAH and soot has been deeply studied mostly in laminar flames as they allow the fine control of the combustion process. *In-situ*^32,33^ [and references therein] and *ex-situ* spectroscopic diagnostics^24,25,34^ have been often applied, exploiting the light absorption and emission properties of soot and PAH. The attribution of spectroscopic signals has required the knowledge of the composition of the carbon particulate matter (soot and organic carbon) sampled in the flame by means of chemical and spectroscopic analysis. It has been found that two- to seven-membered ring PAH justify only a part of the organic species extracted with dichloromethane (DCM) from the carbon particulate matter formed in premixed flames^35^. This inference points towards the presence of heavier aromatic species as supported by size exclusion chromatography (SEC)^36^ and spectroscopic analysis^24,25^. Looking for the heavier aromatic species that are probably involved in soot formation, we have previously discovered the presence of green-fluorescent aromatic species, that were strongly clung to the soot particles. Thus, they had to be stripped with a powerful solvent, namely N-methyl-pyrrolidinone (NMP)^34^. In other very simple combustion systems such as a candle^37^, a pool of a liquid hydrocarbon^38^, and the fumes of waste combustion^39^, green-fluorescent carbon has been produced through the oxidation of carbon soot which was soluble in water. Green-luminescent CDs have also been synthesized from carbon black that was derived from the pyrolysis of bio-based precursors^39–44^. Most of combustion-formed green-fluorescent CDs are water-insoluble^34,38^. Such hydrophobicity can be useful for specific applications as the production of LEDs as well as optoelectronic devices and sensors, where water exposure has to be avoided. Furthermore, the possibility of transforming the combustion-generated CDs into hydrophilic CDs by soot oxidation^37,38^ has to be considered as another advantage of combustion as a source of fluorescent nanoparticles. In particular, laminar flames offer a more suitable environment for a controlled and continuous transformation of carbon/hydrocarbon molecules in CDs of reproducible characteristics.

In this perspective, here we demonstrate how a fuel-rich premixed flame can be used as a reactor for one-step facile and controllable CDs production. Thermophoretic sampling was set up for collecting only soot and condensed phases enriched in the heavier aromatic species and nanoparticles of interest as CDs candidates, avoiding the interference of the smaller hydrocarbons, which escape deposition. Thereafter, the optical (absorption and fluorescence) properties of blue and green fluorescent species separated by sequential solvent extraction of carbon particulate matter have been investigated. Based on the experience gained on soot and PAH in combustion, combined with a preliminary screening, we have focused the analysis on the beginning of the soot formation region of premixed flames burning under fuel-rich conditions. Specifically, the sampling and analysis work has been carried out in flames operating in carbon to oxygen (C/O) molar feed ratio conditions much above the stoichiometric value ((C/O)_stoic_ = 0.33 for ethylene), where the fuel carbon oxidation is incomplete and carbon particulate matter is abundantly formed^24,35,45^.

## Results

**Figure 1:**
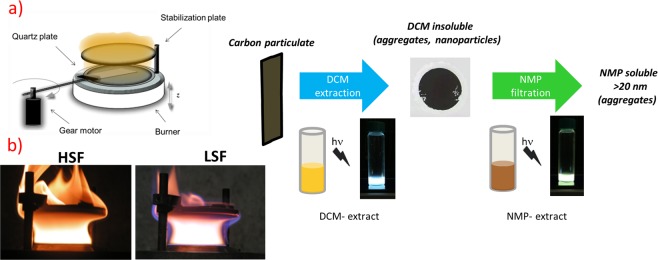
(**a**) Scheme of the experimental setup for carbon particulate matter sampling from premixed flames and solvent treatment, (**b**) photos of the heavily sooting (HSF) and lightly sooting (LSF) reactors.

Carbon particulate matter samples were thermophoretically sampled from a lightly sooting (LSF) and a heavily sooting premixed ethylene flame (HSF). The LSF and HSF flames, produced with a commercial McKenna burner at C/O molar ratios of 0.8 and 1 respectively, were stabilized placing a stabilization plate at 30 mm of flame height. A schematic representation of the experimental setup for carbon particulate matter sampling and treatment along with the flames images are reported in Fig. 1. More experimental details are reported in previous work^35,46^.

**Figure 2:**
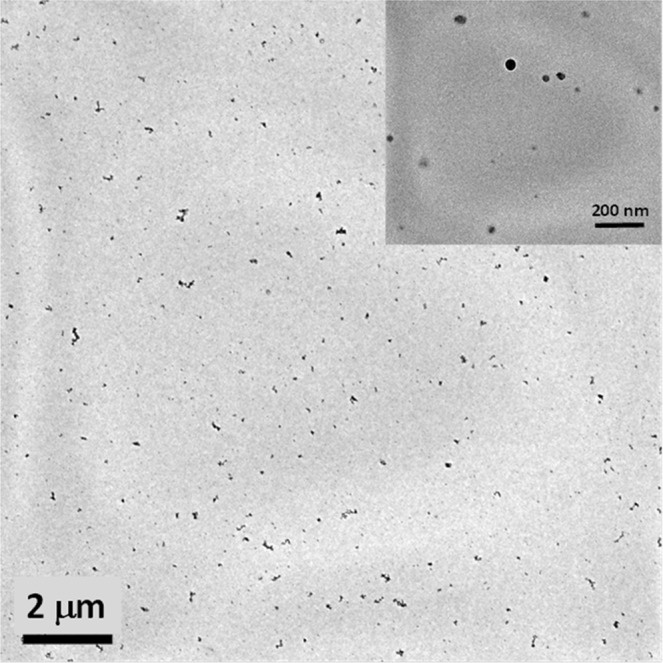
TEM images of flame-synthesized carbon particulate matter.

The condensed phase, heavier than 300 u in molecular weight (MW), was captured via thermophoretic deposition on a rotating quartz plate mounted on an arm and driven by a gear motor. The plate rotation, and consequently the deposition time, was kept to 60 ms per lap. The morphology of carbon particulate matter thermophoretically collected on carbon lacey grids is shown in Fig. 2 reporting the TEM image of a representative carbon particulate matter sample. Both aggregates and individual small-sized CDs around 10 nm, visualized in the inset of the figure, can be noticed.

As noticeable in Fig. 1b, the different intensity of yellow emission related to the soot emissivity highlights the difference in sooting conditions experienced in the HSF and LSF regimes.

The carbon particulate matter caught on the plate was first extracted with DCM and then with NMP to recover all the deposited carbon particulate matter (Fig. 1a). Membrane filters (Anodisc) were used for separating soot particles with diameter >20 nm from NMP dispersions. Both extracts in DCM and NMP were analyzed by UV-Visible absorption and fluorescence spectra (see experimental details in the Methods Section). After a preliminary screening of the maximum excitation and emission wavelength, the fluorescence spectra were recorded to get the emission and excitation spectra, respectively. Synchronous fluorescence spectra were measured while varying simultaneously the excitation and the emission wavelength, but keeping the difference, Δλ, fixed at 10 nm^26,47^. The quantum yield (QY) of the DCM- and NMP-extracts was evaluated at 350 nm by comparison with a standard species (9,10-diphenyl anthracene)^27^.

**Figure 3:**
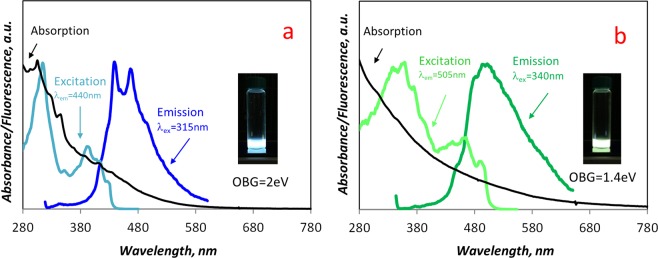
UV-Visible absorption, excitation and emission fluorescence spectra of blue CDs (**a**) (λ_exc_ = 315 nm and λ_em_ = 440 nm), and green CDs (**b**) (λ_exc_ = 340 nm and λ_em_ = 505 nm), extracted from carbon particulate matter sampled at 6 mm of the HSF reactor. The photos of blue and green fluorescent sample solutions and the optical band gap values measured on the UV-Visible spectra are inserted in the respective figures.

The extraction with DCM and successive treatment with NMP of carbon particulate matter, the latter followed by filtration on 20 nm cut filters, produced yellow and brown colored solutions, respectively. These solutions under UV light (365 nm) exhibit fluorescence emission in the blue and in the green wavelength range, respectively, as shown in Fig. 1. This different fluorescence can be also noted in Fig. 3, where the typical excitation and emission spectra of the DCM- and NMP-extracts are displayed in comparison with the relative UV-Visible absorption spectra. The DCM- and NMP-extracts are hereafter named, respectively, blue and green CDs just on the basis of their spectroscopic features below described in greater detail. Table 1 summarises the quantum yield, indicative of the quality, and mass flow rate (quantity) of the blue and green CDs for both HSF and LSF conditions. The mass flow rate of CDs was evaluated referring to a portion of the deposition disk equal to the burner diameter of 60 mm and the total exposure time.

**Table 1:** Quantum yield percentages measured at an excitation wavelength of 350 nm and mass flow rates of blue and green CDs from heavily sooting (HSF) and lightly sooting (LSF) reactors.

Flame Reactor	Flame position mm	Blue CDs		Green CDs	
		quantum yield, % (350 nm)	mass flow rate, mg*h^−1^	quantum yield, % (350 nm)	mass flow rate, mg*h^−1^
HSF	5	24.00	138	2.00	440
	6	21.00	196	5.00	935
	7	20.00	186	6.00	822
LSF	5	17.00	129	3.00	320
	6	19.00	204	3.00	386
	7	20.00	95	4.00	521

The analysis mentioned above has been focused on the carbon particulate matter deposited in the region from 5 to 7 mm of flame height for both HSF and LSF reactors. These flame height positions correspond to the region where soot inception occurs and the formation of organic species reaches the maximum value. The concentration profiles of soot and DCM-extract measured along the axis of HSF and LSF reactors^35^ are reported in the Supplementary Information (Fig. S1). From Fig. S1 it can be observed that the carbon particulate matter formed at the beginning of the soot formation region is mainly constituted of organic species. The enhancing effect of the C/O feed ratio on the mass flow rates of CDs (Table 1), is due to the fuel-rich conditions favoring the formation of light hydrocarbons and carbon particulate matter^35^. The spectra reported in Fig. 3 refer to the blue and green CDs extracted from carbon particulate matter sampled at 6 mm flame height in the HSF reactor. In the following, all figures refer to this sample as this is representative of all the others studied in the present work. Figure 3a clearly shows that the maximum excitation and emission wavelengths of blue CDs (315 and 440 nm) are shifted upward in comparison to green CDs which exhibit the maximum excitation and emission wavelength at 340 and 505 nm, respectively. Overall, it is worth noting that the absorption spectra of both blue and green CDs exhibit the maximum absorption in the UV region while decreasing toward the visible region, which is typical for the π-π absorption of aromatic species. The specific visible absorption peaks, often observed in CDs derived from natural or waste products^22,29^ and attributed to n-π* transitions, were not noticed; their presence, typical of oxygenated aromatics formed in the fuel-rich combustion and from secondary aerosol reactions^48^, was somehow expected.

Figure 3a shows that, for blue CDs, the maximum of the excitation spectrum nearly coincides with the absorption maximum, demonstrating that π−π* absorption of aromatic species is responsible for fluorescence emission. The blue CDs absorption spectrum exhibits a slightly fine-structured shape that is associated with PAH absorption peaks emerging from a π−π* background absorption due to a complex carbon network^49^. The PAH contribution to the blue CDs absorption is due to large PAH (>=C_22_), from 276 to 500 u and peak at 374–398 u (C_30_). This is consistent with the mass spectrometric profile measured by laser desorption-time of flight-mass spectrometry of blue CDs (Supplementary Information Fig. S2). Accordingly, also the emission spectrum of blue CDs (Fig. 3a) is slightly structured, showing two main peaks in the region where large PAH fluoresce^24,50^. The optical band gap (OBG) of 2 eV, evaluated by Tauc analysis of the UV-Visible spectrum of blue CDs^51^, is sufficiently high to allow considerable light emission, as shown in Table 1 (quantum yield around 20%). Furthermore, this is consistent with the nanometric-subnanometric size of large PAH.

As mentioned above, the green fluorescence from carbon particulate matter samples is typical for species that are strongly absorbed on soot and can only be stripped with powerful solvents such as NMP^34,51^. The use of DCM, cyclohexane, ethanol, and water as solvents was attempted, however, these solvents were not able to achieve solubilization with the green CDs. Compared to the blue CDs (Fig. 3a), the green CDs exhibit a broader absorption spectrum with a tail extended into the visible wavelength range (Fig. 3b). Consistently, the green CDs present a lower optical band gap (1.4 eV), corresponding to a lower quantum yield (2–6%) (Table 1). Furthermore, the maximum of the excitation spectrum is shifted toward the visible range (400 nm) with respect to the UV absorption maximum (<280 nm) (Fig. 3b), indicating that only the visible absorbing chromophores featuring the green CDs are able to emit fluorescence. The broad shape of the emission spectrum observed in Fig. 3b is typical for green CDs^10^, neither attributable to n−π* transitions nor to individual fluorophore molecules, but mainly ascribable to PAH domains featuring macromolecular carbonaceous material. It is worth underlining that the fluorescence of organic carbon, directly extracted as a whole from carbon particulate matter with NMP, was found to be dominated by the blue fluorescent molecules. Blue CDs overcome and mask the green carbon fluorescence measured because of their much higher quantum yield and the partial overlapping of blue and green fluorescence spectra. Actually, the details of how blue and green CDs show to grade one into the other will be covered in the following part of the work which focuses on size and/or molecular weight analysis.

**Figure 4:**
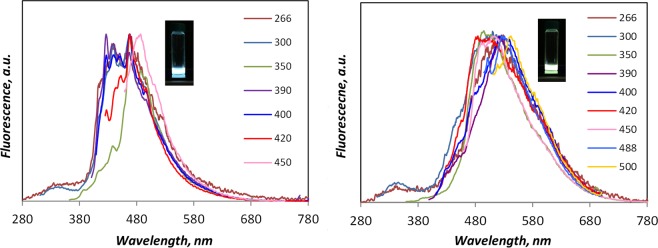
Normalized fluorescence spectra of blue CDs (left) and green CDs (right) excited at different wavelengths. The photos of blue and green fluorescent sample solutions are inserted in the respective figures. The reported spectra refer to CDs sampled at 6 mm of flame height in heavily sooting (HSF) reactor.

The emission spectra of blue and green CDs measured at different excitation wavelengths are displayed in Fig. 4. In the case of blue CDs (left panel of Fig. 4) a downward shift of the maximum emission is observed at an excitation wavelength (350 nm), corresponding to the deep valley separating the first and second region of the excitation spectrum, respectively peaked at 320 and 380 nm (Fig. 3a). This behavior, as well as the fine structure pattern, can be attributed to the significant presence in the blue-CDs of individual fluorescent molecules as large PAH distributed in the 300–500 u mass range (Fig. S2). Unlike the blue CDs, the almost excitation-independent spectra of the green CDs (right panel of Fig. 4) suggest their homogenous macromolecular nature also indicated by the broad UV-Visible spectra and consistent with the bigger size of green CDs^39^.

**Figure 5:**
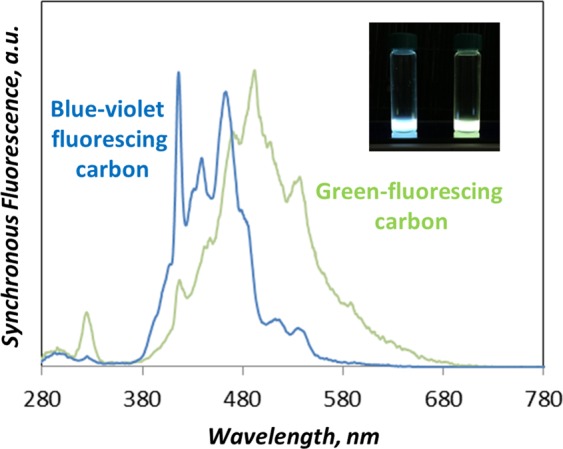
Synchronous emission spectra (Δλ = 10 nm) of blue and green CDs extracted from carbon particulate matter sampled at 6 mm of flame height in heavily sooting (HSF) reactor. The photos of blue and green fluorescent sample solutions are inserted in the figure.

Synchronous fluorescence, measured by applying a wavelength difference of 10 nm between excitation and emission wavelengths^26,47^, is an additional fluorescence-based tool, useful to discriminate between classes of different-sized PAH, thus providing further insights on blue and green CDs features. Consistently with the absorption and emission features (Fig. 3), the synchronous spectrum of blue CDs, reported in Fig. 5, exhibits few well-defined peaks due to large PAH^26,52^, whereas the synchronous spectrum of the green CDs displays a broader shape extended and peaked downward. Remarkably, the synchronous spectra of blue and green CDs (Fig. 5) clearly show some overlapping of blue CDs fluorescence peak tail with the head of green CDs fluorescence peak. On the basis of the spectroscopic properties described above, blue and green CDs should exhibit different size and/or molecular weight, even though some reciprocal contamination is suggested by the fluorescence spectra overlapping (Fig. 5).

**Figure 6:**
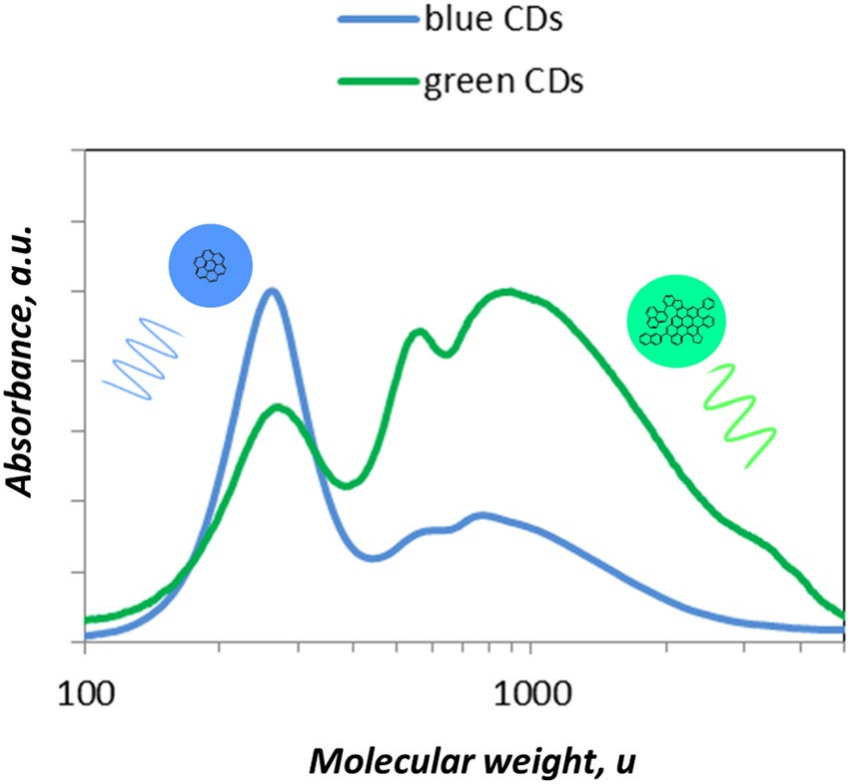
Molecular weight distribution of the blue and green CDs as measured by SEC analysis. The reported distributions refer to the CDs sampled at 6 mm of the HSF reactor.

The extent to which blue and green CDs are separated by DCM and NMP extraction can be evaluated by the separation in size-, MW-segregated fractions^36^ obtained by SEC analysis coupled with UV-Visible absorption detector fixed at 350 nm. Figure 6 shows a typical MW/size distribution of blue and green CDs. It can be seen that the MW distribution of blue CDs peaks around 400 u, consistently with the mass spectrometric analysis (Fig. S2).

The MW distribution of the green CDs is mostly localized on the opposite side of the MW range, peaking around 10^3^ u and broadening toward the 10^4^ u limit. The MW distributions of the blue and green CDs show to grade continuously one into the other in agreement with the partial overlapping of their fluorescence spectra above described (Fig. 5). It is remarkable that the distribution of blue and green CDs, in the 100 to 10^4^ u MW range, nearly corresponds to the size range, from subnanometric to nanometric (up to about 2 nm), by assuming spherical particles with an average density of 1.5 g/cm^3^. The optical band gap evaluated on the UV-Visible spectrum measured on-line to the SEC system, namely on the peak apex, decreased from about 2.0 eV for the main peak of the blue CDs, down to 1.5 eV for the main peak of the green CDs, consistently with the band gap evaluated on the UV-Visible spectra of the as-prepared green and blue CDs (Fig. 3).

More information on the size and structure of green CDs was obtained in previous work concerning the fluorophores size around 1.5 nm as evaluated by measuring rotational times (around 1 ns) by time-resolved fluorescence polarization anisotropy (TRFPA)^53,54^. The blue CDs fluorophore size could not be measured because the rotational times were too fast («1 ns) to be detected with the experimental apparatus used. Interestingly, the green CDs rotational times were found to be independent on the emission wavelength in the 350–600 nm emission range^53^. The absence of a correlation between the TRFPA diameter, related to the molecular size, and the emission wavelength, related to the chromophore size, suggests that the green fluorescence is not due to individual large fluorophore molecules, but to a limited variety of not very big chromophores (around 10–20 aromatic condensed rings), bonded and/or turbostratically stacked together. Although disentangling the origin of fluorescence from CDs and the attribution to molecules or to aggregates/particles is still very challenging and debated^15,29,55–57^, the fluorescence of flame-formed CDs appears to be due to the carbon aromatic core. This is demonstrated by the general predominance of aromatic moieties, the absence of surface functionalities along with the excitation-independent emission. Specifically, with regards to the structure and origin of green CDs fluorescence, the inferences so far obtained hint to PAH domains featuring macromolecular carbonaceous material, responsible for green fluorescence, whereas some contributions of free or surface-linked fluorescent PAH molecules appear rather significant as regards blue CDs fluorescence origin.

Overall, it has been shown that premixed fuel-rich flames are a suitable reactor for the synthesis of hydrophobic blue and green CDs, presenting an almost excitation-independent luminescence and well-defined features. Both the scalability of flame reactors and the capacity to stably and continuously produce CDs of reproducible characteristics make them a promising alternative to other bottom-up processes which involve harsh and time-consuming treatment of precursors. Aside the possibility of transforming combustion-formed CDs into hydrophilic CDs by oxidation^37,38^, green and blue CDs can also be easily used for applications requiring the CD’s hydrophilicity by diluting the NMP solutions of CDs with water, thanks to the complete miscibility of NMP with water. Concerning the effect of C/O feed ratio on the production and quality of flame-formed CDs, summarized in Table 1, it can be noticed that the higher C/O feed ratio of highly sooting flames, as the HSF reactor, favors green CDs formation. Moreover, in the interval of the C/O ratio investigated, it appears that the C/O ratio affects the yield of CDs without changing their quantum yield. The optimal conditions of blue and green CDs synthesis occur at 6 mm of flame height, i.e. in the middle of soot inception region, where the QY is higher and the mass flow rate is maximum. It is foreseen that higher C/O ratios, i.e. fuel-richer conditions, and/or a decrease of flame temperature, could further enhance CDs production by quenching the reactions which lead toward soot formation.

## Methods

The absorption spectra of the samples were recorded on a diode array spectrophotometer (HP Agilent 8453) in the 260–800 nm wavelength range. The steady-state fluorescence spectra were recorded in the 220–700 nm wavelength range with a spectrofluorometer (Perkin-Elmer LS-50). Size exclusion chromatography (SEC) of DCM- and NMP extracts was carried out on a HPLC system equipped with a diode array UV-Visible detector. The samples were eluted with NMP on a PL-gel styrene-divinylbenzene individual pore column (Polymer Laboratories, Ltd, UK) with a column particle size of 5 μm diameter and a pore dimension of 50 nm. This column is able to separate polystyrene standards in the molecular mass range from 100 to 10^4^ u (corresponding to sizes of about 2 nm). TEM in lattice fringe mode was performed on the soot samples using a JEOL 2011 electron microscope operating at 200 kV equipped with a LaB6 filament (resolution in the lattice fringe mode 0.144 nm). Carbon particulate matter was thermophoretically sampled directly on the carbon lacey grid.

## Supplementary information

Supplementary information
