# Supplementary material for: Blue and green luminescent carbon nanodots from controllable fuel-rich flame reactors
Source: Sci Rep. 2019 Oct 10;9:14566. doi: 10.1038/s41598-019-50919-1 (PMC6787054; doi:10.1038/s41598-019-50919-1)
Supplement: Supplementary file 1 — Supplementary information [file 41598_2019_50919_MOESM1_ESM.doc]

**Blue and green luminescent carbon nanodots**

**from controllable fuel-rich flame reactors**

*Carmela Russo, Barbara Apicella, Anna Ciajolo**

Istituto di Ricerche sulla Combustione, Consiglio Nazionale delle Ricerche, Piazzale V. Tecchio 80, 80125 Napoli.

Corresponding Author

*E-mail: ciajolo@irc.cnr.it

**Supplementary information of the paper:**

Blue and green luminescent carbon nanodots from controllable fuel-rich flame reactors

1. ***Concentration profiles of carbon particulate matter***

The concentration profiles of carbon particulate matter distinguished in soot and organic carbon as sampled in the LSF and HSF flame are reported in Fig. S1. The data reported in the figure are from Ref. [1].

Fig. S1 Concentration profiles of carbon particulate matter distinguished in soot and organic carbon as sampled in the LSF (in red) and HSF (in black) flame.

1. ***Laser desorption ionization time of flight mass spectrometry (LDI-TOFMS) of carbon particulate matter***

Laser Desorption Ionization-Time-of-Flight Mass Spectrometry spectra were recorded on positive reflectron mode on a AB SCIEX TOF/TOF™ 5800 System. The target was prepared by depositing on the metallic sample plate a volume variable from 1 and 10 μl of a solution of the sample dispersed in DCM. Matrices were not added as all of the investigated samples are able to absorb the laser beam (λ = 337 nm) acting as a self-matrix.

The mass spectrometric profile measured by LDI-TOFMS of blue-CDs sampled in the HSF flame at 6 mm of height above the burner is reported in Fig. S2. It is possible to observe the main presence of large PAH (>C_24_) from 300 to 500 u peaked at 374-398 u (C_30_). The spectrum is dominated by two sequences (with a m/z 12 spacing between them) of higher (masses in black) and lower (masses in red) intensities with incremental gaps of m/z 24-26, attributed to even- and odd- numbered PAH [2].

Fig. S2. LDI-TOFMS of blue-CDs sampled in the HSF flame at 6 mm of height above the burner.

**References**

(1) Apicella, B.; Barbella, R.; Ciajolo, A.; Tregrossi, A. Formation of low and high molecular weight hydrocarbon species in sooting ethylene flames. Combust. Sci. Tech., 2002, 174 (11-12), 309-324, DOI: 10.1080/713712948.

(2) Panariello, M.; Apicella, B.; Armenante, M.; Bruno, A.; Ciajolo, A.; Spinelli, N. Analysis of polycyclic aromatic hydrocarbon sequences in a premixed laminar flame by on-line time-of-flight mass spectrometry. *Rapid Comm. in Mass Spectrom.* **2008,**  *22 (4),* 573-581. DOI: 10.1002/rcm.3391.
